# Mild Motor Signs Matter in Typical Brain Aging: The Value of the UPDRS Score Within a Functionally Intact Cohort of Older Adults

**DOI:** 10.3389/fnagi.2021.594637

**Published:** 2021-02-11

**Authors:** Jennifer Zitser, Kaitlin B. Casaletto, Adam M. Staffaroni, Claire Sexton, Sophia Weiner-Light, Amy Wolf, Jesse A. Brown, Bruce L. Miller, Joel H. Kramer

**Affiliations:** ^1^Memory and Aging Center, University of California, San Francisco, San Francisco, CA, United States; ^2^Global Brain Health Institute, University of California, San Francisco, San Francisco, CA, United States; ^3^Movement Disorders Unit, Department of Neurology, Tel Aviv Sourasky Medical Center, Affiliated to the Sackler Faculty of Medicine, Tel-Aviv University, Tel Aviv-Yafo, Israel

**Keywords:** aging, parkinsonism, UPDRS, cognition, fMRI, motor signs

## Abstract

**Objectives:** To characterize the clinical correlates of subclinical Parkinsonian signs, including longitudinal cognitive and neural (via functional connectivity) outcomes, among functionally normal older adults.

**Methods:** Participants included 737 functionally intact community-dwelling older adults who performed prospective comprehensive evaluations at ~15-months intervals for an average of 4.8 years (standard deviation 3.2 years). As part of these evaluations, participants completed the Unified Parkinson's Disease Rating Scale (UPDRS) longitudinally and measures of processing speed, executive functioning and verbal episodic memory. T1-weighted structural scans and task-free functional MRI scans were acquired on 330 participants. We conducted linear mixed-effects models to determine the relationship between changes in UPDRS with cognitive and neural changes, using age, sex, and education as covariates.

**Results:** Cognitive outcomes were processing speed, executive functioning, and episodic memory. Greater within-person increases in UPDRS were associated with more cognitive slowing over time. Although higher average UPDRS scores were significantly associated with overall poorer executive functions, there was no association between UPDRS and executive functioning longitudinally. UPDRS scores did not significantly relate to longitudinal memory performances. Regarding neural correlates, greater increases in UPDRS scores were associated with reduced intra-subcortical network connectivity over time. There were no relationships with intra-frontoparietal or inter-subcortical-frontoparietal connectivity.

**Conclusions:** Our findings add to the aging literature by indicating that mild motor changes are negatively associated with cognition and network connectivity in functionally intact adults.

## Introduction

Mild Parkinsonian signs -including rigidity, bradykinesia, tremor, or posture and gait disturbance- are often present on the clinical examination of healthy older adults who do not fulfill criteria for Parkinson's disease (PD) or other neurodegenerative diseases (Kokmen et al., 1977; Funkenstein et al., 1993; Kaye et al., 1994; Bennett et al., 1996). The prevalence of such signs continues to increase with age, in fact, up to half of community dwelling adults aged 85 years and older demonstrates Parkinsonian signs on examination without a clinical diagnosis of PD (Bennett et al., 1996). Yet, the true meaning and clinical importance of these motor changes in the “healthy” aging population are not well-understood.

These mild Parkinsonian signs have been associated with increased mortality and other negative health outcomes including falls (Bennett et al., 1996; Louis and Bennett, 2007; Buracchio et al., 2010), functional disability, and loss of independence (Louis et al., 2006; Fleischman et al., 2007). Nonetheless, little is known about their underlying anatomical circuitry, or whether they are associated with changes in cognition in functional intact elders.

In PD, disrupted basal ganglia circuits are the hallmark neural correlate of these motor signs and cognitive changes. In fact, the severity of motor signs often tracks closely and is a risk factor for development of the slowed processing speed and mild executive dysfunction evident in PD (Siciliano et al., 2017). Additionally, several task-free (“resting state”) functional magnetic resonance imaging (tf-fMRI) studies have demonstrated that functional connectivity is reduced within the basal ganglia of patients with PD compared to patients with Alzheimer's disease (AD) or healthy controls (Rolinski et al., 2015), and basal ganglia connectivity increases upon administration of dopaminergic medication (Szewczyk-Krolikowski et al., 2014). Also, basal ganglia connectivity correlates with clinical indices of disease severity, including PD-related cognitive decline (Tuovinen et al., 2018). Nevertheless, it is unclear if in a population of functionally intact aging adults, mild Parkinsonian signs are also associated with these or similar hallmarks. Careful characterization of the clinical correlates of these very common age-related but mild motor changes would significantly enhance both pathophysiologic understanding of Parkinsonian signs and guide recommendations in the clinic environment.

In the current study, we characterized the longitudinal cognitive and neural correlates of Parkinsonian signs among functionally normal older adults. Based on the cognitive profile of PD patients, we hypothesized that these mild Parkinsonian signs would track with particular cognitive domains known to be affected in clinical PD (i.e., processing speed and executive function) and with mood. We also hypothesized that longitudinal increases in UPDRS scores would track with declines in basal ganglia related connectivity. Identification of such a relationship across time has potential clinical implications, forcing us to challenge the importance of seemingly “non-specific” motor exam findings for the functionally intact elderly.

## Materials and Methods

### Participants

For this study, we included 737 community-dwelling older adults (baseline ages 55–99 years) enrolled in clinical research at the University of California, San Francisco Memory and Aging Center (UCSF). Participants were followed as part of the Hillblom healthy aging study, which is an ongoing study with continued recruitment, where participants complete comprehensive evaluations at ~15-months intervals for an average of 4.8 years (standard deviation 3.2 years; range of time points 1–11, depending on when the participant was recruited), including neurological exams, brain MRI, and neuropsychological assessment. Only participants that were functionally intact, demonstrated by a score of 0 on the Clinical Dementia Rating (CDR) scale via interviews with study partners, completed the UPDRS, had no major memory concerns or diagnosed memory condition, and did not meet consensus criteria for any neurodegenerative disease [Parkinson's disease (PD), Dementia with Lewy bodies (DLB), Multi-system Atrophy (MSA), progressive supranuclear palsy (PSP), Alzheimer's disease, behavioral variant frontotemporal dementia (bvFTD) and semantic variant primary progressive aphasia (svPPA)] *at all study visits*, were included in the current analyses. For as long as the selected participants were followed in our study, none converted to a Parkinsonian-related or other neurodegenerative syndrome.

### Standard Protocol Approvals, Registrations, and Patient Consents

The University of California San Francisco Institutional Review Board (IRB) approved this study and informed written consent was obtained from all participants.

### Parkinsonian Signs

The Unified Parkinson's Disease Rating Scale (UPDRS) is the gold standard clinical rating scale for Parkinsonism and was completed by board-certified neurologists trained to administer the measure (Movement Disorder Society Task Force on Rating Scales for Parkinson's Disease, 2003). In this study only UPDRS part III (motor evaluation) was performed. The UPDRS Motor Examination is a structured neurologic examination that includes 27 items with scores ranging from 0 (normal) to 4 (severely impaired) for a maximum total score of 108 points. Each item is score individually.

### Cognitive Assessment

#### Information Processing Speed

Cognitive assessment methods were previously described elsewhere (Casaletto et al., 2018). All participants were administered a set of four computerized tasks evaluating processing speed (single lexical decision, double lexical decision, category judgment, antonym-synonym judgment) (Lawrence et al., 1998). On single lexical decision, participants determined whether a string of consonant-vowel-consonant letters comprised of high frequency (e.g., top), low frequency (e.g., rib), or non-word (e.g., ged) is a word in English. On double lexical decision, participants were presented with two strings of letters (i.e., related word pairs, unrelated word pairs, non-word–word pairs, and word–non-word pairs) and needed to determine if both were words in English. In category judgment, participants determined if two words were from the same semantic category, and in antonym-synonym, participants decided if the meaning of two words were the same or opposite. All tasks had a practice trial period where the subject had to perform at >70% accuracy to continue to the test trials (Kerchner et al., 2012). Task performances were combined into a single reaction time composite score with higher scores reflecting poorer performance (slower reaction time).

#### Executive Functions

We administered the NIH EXAMINER (Kramer et al., 2014). We obtained a composite score from five computer-based tests: two verbally-mediated tests of generativity (Lexical and Animal fluency), one of working memory (Dot Counting, 1-Back, 2-Back), one of response inhibition (Enclosed Flanker), and one of set shifting (Set Shifting).

#### Episodic Memory

We administered the California Verbal Learning Test-second edition (CVLT-II) (Woods et al., 2006). Participants were asked to freely recall the 16-item list after an interference trial, and again after a 20–30-min delay. Our primary memory outcome was free recall after 20–30 min (possible range 0–16 words).

### Depressive Symptoms

We administered the Geriatric Depression Scale (GDS), a 30-item self-report screener of depressive symptoms for older adults (Yesavage et al., 1982). Each question is scored 0 or 1 point, with a score of 0–10 considered normal/minimal, 11–20 moderate, and 21–30 severe depression.

### Neuroimaging

#### Acquisition

**Figure 1:**
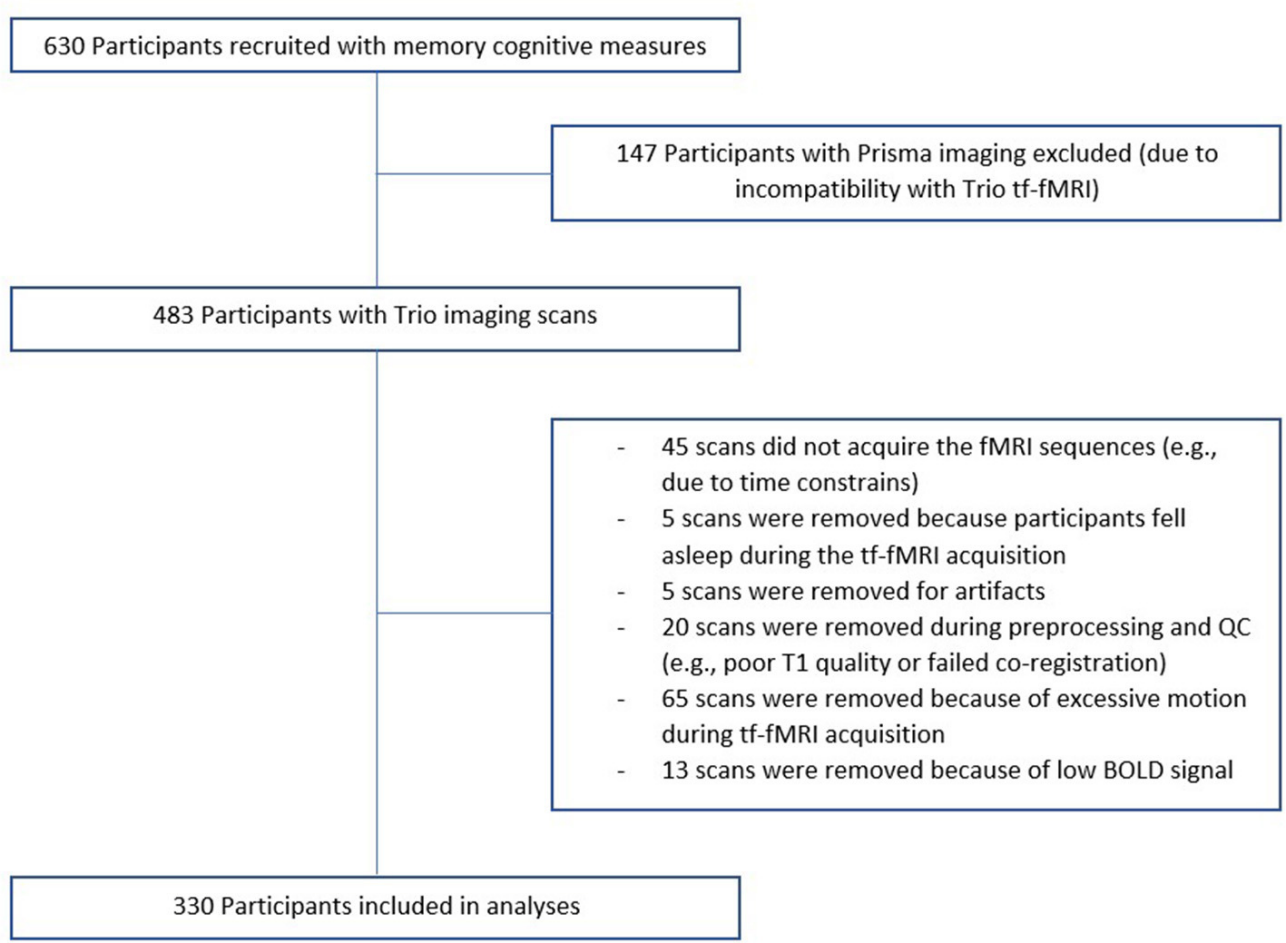
Recruitment flowchart. Of our 737 participants, 630 had memory cognitive measures. 483 were scanned at the UCSF Neuroscience Imaging Center on a Siemens Trio 3T scanner and 330 subjects were considered for analyses.

Of our 737 participants, 483 were scanned at the UCSF Neuroscience Imaging Center on a Siemens Trio 3T scanner and 330 subjects were considered for analyses (see Figure 1 for details on the reasons for exclusion). A T1-weighted MP-RAGE structural scan was acquired with an acquisition time = 8 min 53 s, sagittal orientation, a field of view of 160 × 240 × 256 mm with an isotropic voxel resolution of 1 mm^3^, TR = 2,300 ms, TE = 2.98 ms, TI = 900 ms, flip angle = 9°. Task-free T2^*^-weighted echoplanar fMRI scans were acquired with an acquisition time = 8 min, 6 s, axial orientation with interleaved ordering, field of view = 230 × 230 × 129 mm, matrix size = 92 × 92, effective voxel resolution = 2.5 × 2.5 × 3.0 mm, TR = 2,000 ms, TE = 27 ms, for a total of 240 volumes. During tf-fMRI acquisition, participants were asked to close their eyes and concentrate on their breath.

Methodology for longitudinal T1 processing can be found in the Supplementary Material 1.

#### fMRI Pre-processing

fMRI processing and network construction has been previously described (Staffaroni et al., 2018) and details are provided in the Supplementary Material 2. Regions with insufficient fMRI BOLD signal to noise ratio were excluded using a previously described procedure (Staffaroni et al., 2018). Based on this procedure, we excluded 49 scans and 45 regions (which were not entered into the dataset). Further detail can be found in the Supplementary Material 2, and information about selection of scans is presented on Figure 1.

#### Network Construction

Functional networks were defined in a data-driven fashion using a single scan per person in a set of 75 functionally intact older control participants (our “HC2” group; mean age = 65.3 ± 10.0 years, 33 females/42 males, mean education = 17.3 ± 2.1 years, 68 right handed/7 left handed); using the same pipeline as the participants in the longitudinal portion of this study, these subjects were scanned and analyzed. The details of our process have been described elsewhere (Staffaroni et al., 2018). In summary, we utilized a modularity-based method for identifying which nodes comprised each module -intrinsic connectivity network-, adopting a similar strategy to that used by Power et al. (2011), implementing the Brain Connectivity Toolbox (https://sites.google.com/site/bctnet/). Twenty seven of 75 of the HC2 controls overlap with the participants used in the primary analysis.

**Figure 2:**
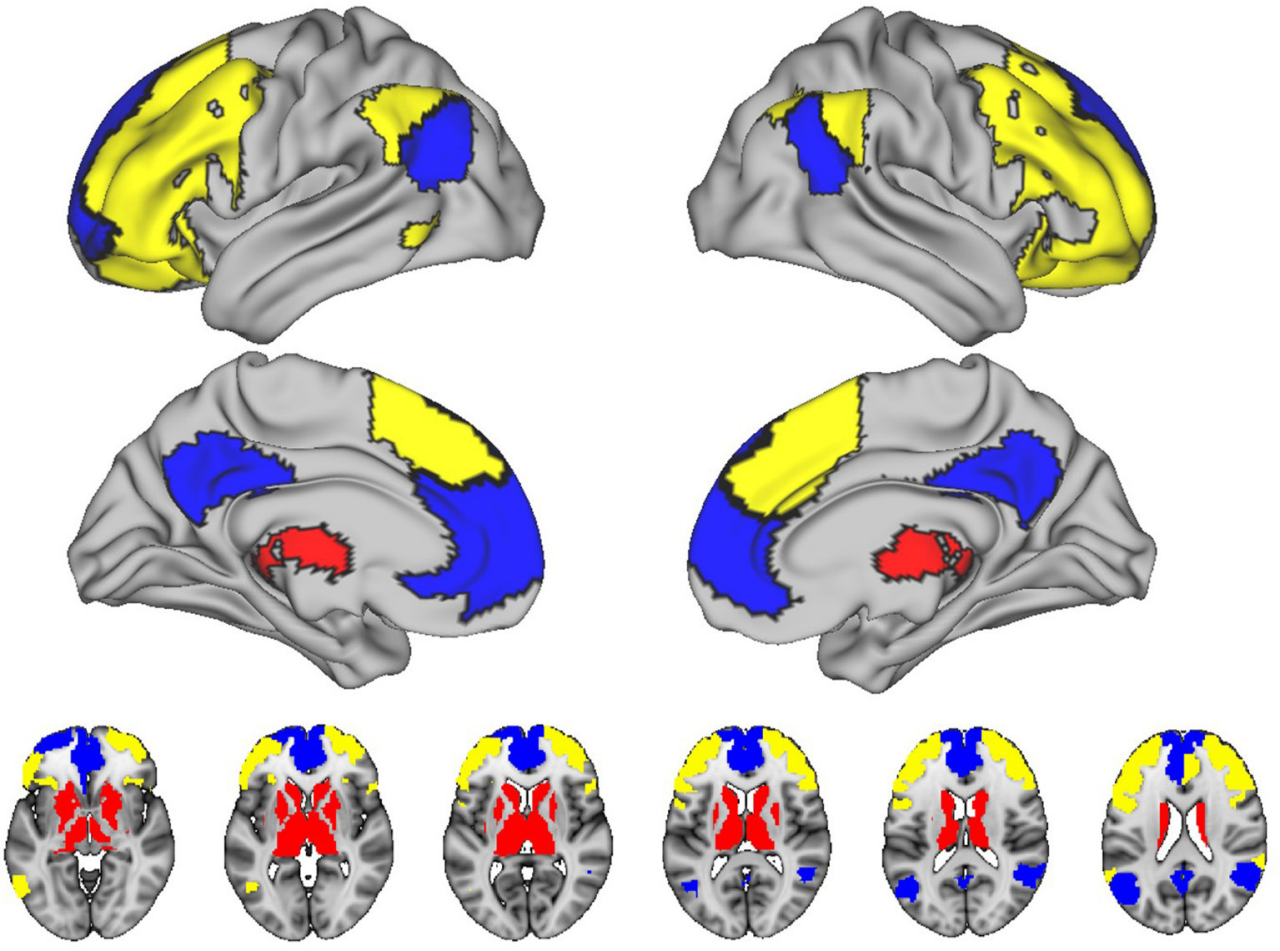
Networks included in this study. The figure shows the connectivity within three networks known to be related to cognitive aging: the default mode (DMN), frontoparietal, and subcortical networks. Blue, default mode; Yellow, left/right executive; Red, subcortical.

In HC2 subjects, we determined the whole brain functional connectome using 228 regions from the Brainnetome atlas and SUIT atlas (Diedrichsen, 2006; Fan et al., 2016). In order to reduce the number of networks, and therefore the number of statistical comparisons, we focused on connectivity within three networks known to be related to cognitive aging: the default mode (DMN), frontoparietal, and subcortical networks, consisting of the basal ganglia and thalamus (see Figure 2) (Campbell et al., 2012; Ousdal et al., 2020). We also studied the connectivity between the frontoparietal and subcortical networks, which we recently have shown is germane to cognitive aging. We calculated four mean functional connectivity values for each participant. Using the unthresholded matrices we calculated the mean of the edges between all nodes within or between networks: intra-DMN, intra-frontoparietal, intra-subcortical, and subcortical-frontoparietal (between subcortical and frontoparietal networks). Whole-brain FC was calculated by taking the mean of the edges between all nodes in the brain that were not excluded, without threadholding.

Brainnetome regions comprising these tf-fMRI networks were then applied to the T1 scans in order to extract gray matter volumes. The regional volumes for all regions comprising a given network were summed (and divided by total intracranial volume) in order to obtain the network's gray matter volume.

### Data Availability Statement

Raw data that supports the findings of this study were generated at the Memory and Aging Center (MAC) at UCSF. Derived data supporting the findings of this study are available from the corresponding author on request.

### Statistical Analysis

To characterize the longitudinal trajectories of Parkinsonian signs in our typically aging cohort, we conducted a series of linear mixed-effect regression models. Covariates in all models included baseline age, sex, and education, and mixed effects models allowed for subject specific intercepts and slopes. First, we evaluated changes in Parkinsonian symptoms by evaluating the relationship between study time (years) and UPDRS. Next, we specifically probed the effect of age on the relationship between study time and UPDRS by introducting the interaction between baseline age^*^time as the independent variable and UPDRS as the dependent variable. This latter model aimed to determine if the slope of UPDRS trajectories significantly changed (e.g., increased or decreased) differentially by age.

To determine the relationship between changes in UPDRS score and cognitive and neural changes, we first decomposed UPDRS into within- and between-subject components, following Neuhaus and Kalbfleisch (1998); this was done in order to associate within-subject changes in UPDRS with changes in cognitive and fMRI outcomes, and to avoid estimation bias resulting from incorrectly assuming common within- and between-subject effects. Next, we conducted linear mixed effects regression models entering in both the UPDRS within- and between-subject terms, as well as relevant covariates (baseline age, sex, and education) as our independent variables and cognition or fMRI network as the dependent variable. All models were *a priori* specified based on our hypothesized relationship between UPDRS and cognitive or fMRI outcomes. In fMRI models, we conducted sensitivity models further adjusting for whole brain connectivity, as well as models with whole brain connectivity and the volume of that region.

## Results

**Figure 3:**
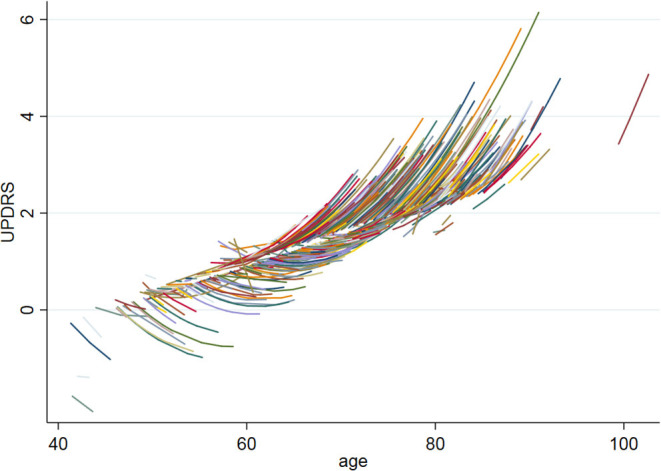
UPDRS baseline age by time interaction. The figure shows the UPDRS baseline age by time interaction, such that the slope of UPDRS scores was negative in adults <60 years old and became significantly more positive with subsequent increasing age (baseline age*time: *b* = 0.009, *p* = 0.03).

In our cohort of community-dwelling functionally intact adults, the mean MMSE score was 29.2 and the mean UPDRS scores was 1.4. UPDRS scores significantly increased over time (*b* = 0.07, *p* = 0.03) and with age (*b* = 0.10, *p* < 0.001). In fact, there was a baseline age by time interaction, such that the slope of UPDRS scores was negative in adults <60 years old and became significantly more positive with subsequent increasing age (baseline age^*^time: *b* = 0.009, *p* = 0.03) (see Figure 3). The demographic characteristics of our population are depicted in Table 1.

**Table 1:** Baseline demographic characteristics.

	**Cognitive measures**	**fMRI measures**
Sample size	737	330
Age, years (mean and standard deviation)	67.5 (±11.4)	70.47 (±7.6)
Sex, % female	422 (58.37%)	123 (54.91%)
Education, years (mean and standard deviation)	17.07 (±2.18)	17.26 (±2.14)
**Number of visits**		
1	737	330
2	359	247
3	230	114
4	172	67
≥5	127	62
UPDRS	1.46 (2.5) (range: 0, 20)	1.31 (2.3) (range: 0, 20)
GDS	3.3 (3.6)	2.8 (3.0)
Processing speed (z-score)	1.5 (1.1)	1.4 (1.2)
Executive functions (z-score)	0.87 (0.60)	0.88 (0.60)
Episodic Memory (CVLT-II Delayed Recall, words)	11.5 (3.1)	11.6 (3.1)

*UPDRS, Unified Parkinson's Disease Rating Scale; GDS, Geriatric Depression Scale; CVLT-II, California Verbal Learning Test Second Edition. Data are mean and standard deviation (SD) unless otherwise indicated*.

### Cognitive Outcomes

**Figure 4:**
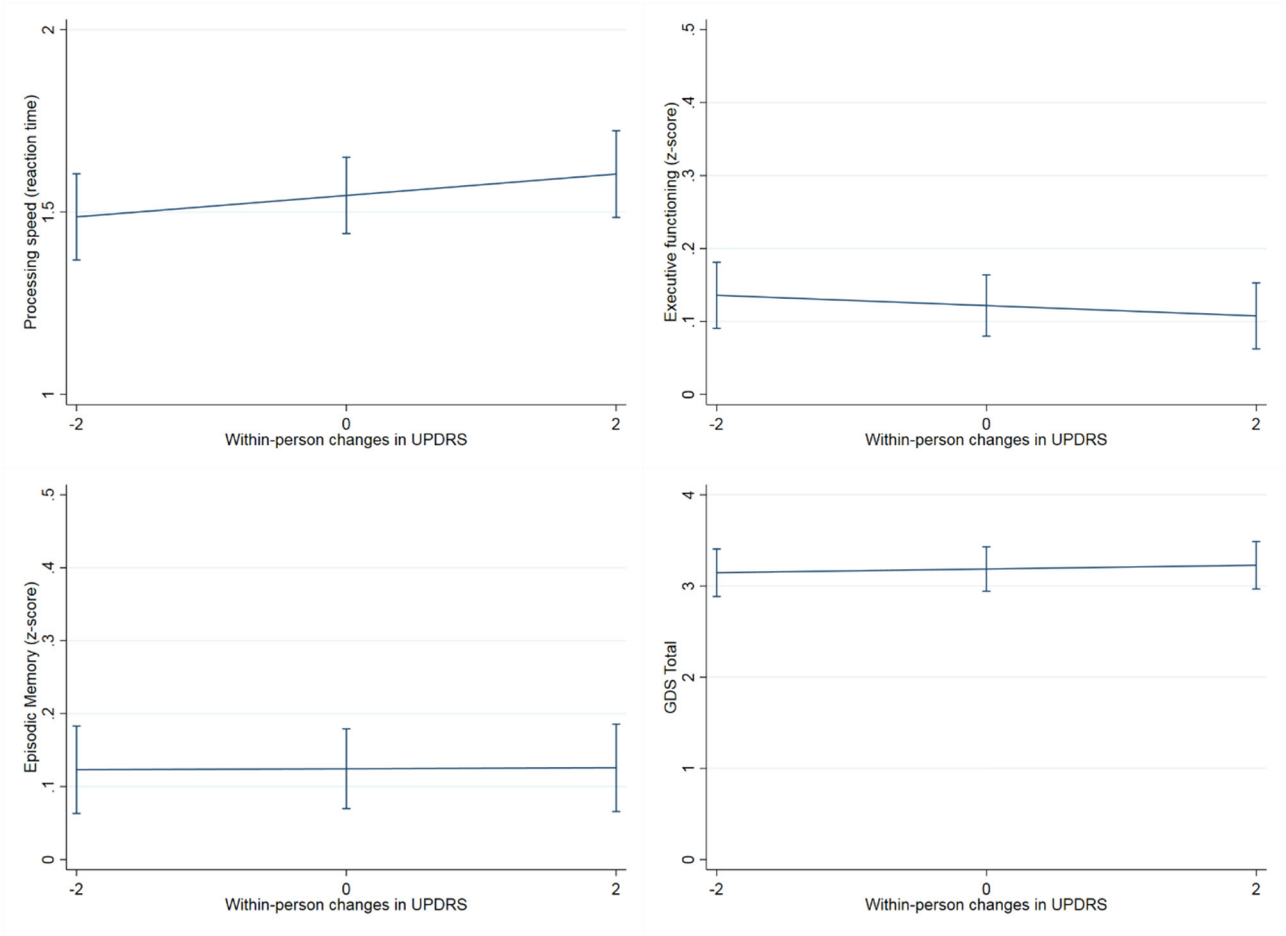
Association between within-person changes in UPDRS scores and cognitive measures. The figure shows how within-person increases scores tracked with processing speed slowing over time (years), but not with episodic memory, executive functioning or mood (GDS: Geriatric depression scale). Processing speed was measured with a set of four computerized tasks (single lexical decision, double lexical decision, category judgment, antonym-synonym judgment), task performances were combined into a single reaction time composite score with higher scores reflecting poorer performance (slower reaction time). Executive functioning was tested through a composite score from five computer-based tests of working memory (Dot Counting, 1-Back, 2-Back), response inhibition (Enclosed Flanker), and set shifting (Set Shifting), and two verbally-mediated tests of generativity (Lexical and Animal fluency). Finally, Episodic memory was measured with the California Verbal Learning Test-second edition (CVLT-II; z score).

Within-person increases in UPDRS scores tracked with significant within-person cognitive slowing over time (UPDRS within-person *b* = 0.032, *p* = 0.03, UPDRS between-person *b* = 0.07, *p* = 0.005) (Figure 4). Overall higher UPDRS scores were associated with worse executive functions (overall/between-person *b* = −0.033, *p* < 0.001) and mood (overall/between-person *b* = 0.12, *p* = 0.03), though the within-person UPDRS terms in both of these models did not reach statistical significance. These latter models suggest that although motor symptoms correlate with executive functions and mood, these factors do not significantly co-vary together with each other across time. UPDRS scores were not significantly associated with memory performances trajectories (overall/between-person *b* = −0.009, *p* = 0.44; within-person *b* = −0.003, *p* = 0.62) (Table 2).

**Table 2:** Within person and Between person UPDRS scores and cognitive variables correlations in a cohort of functionally intact community-dwelling older adults.

	**Sample size (number of observations)**	**Within person**		**Between person**	
		**Coefficient (95% CI)**	***p*-Value**	**Coefficient (95% CI)**	***p*-Value**
Processing speed	405 (711)	0.0294 (0.0017, 0.0571)	0.038	0.0708 (0.0244, 0.1173)	0.003
Executive functions	737 (1,767)	−0.0071 (−0.0154, 0.0013)	0.097	−0.0411 (−0.0579, −0.0244)	0.000
Memory	630 (1,544)	0.0006 (−0.0116, 0.0129)	0.917	−0.013 (−0.0351, 0.0092)	0.251
Mood	717 (1,706)	0.0205 (−0.026, 0.0669)	0.387	0.0992 (0.0001, 0.1983)	0.05

*Within person and between person [and 95% Confidence Interval (CI)] are shown. Overall higher UPDRS scores were associated with worse executive functions (overall/between-person b = −0.033, p < 0.001) and mood (overall/between-person b = 0.12, p = 0.03)*.

*Covariates in all models included baseline age, sex, and education, and mixed effects models allowed for subject specific intercepts and slopes*.

### fMRI Connectivity Outcomes

**Figure 5:**
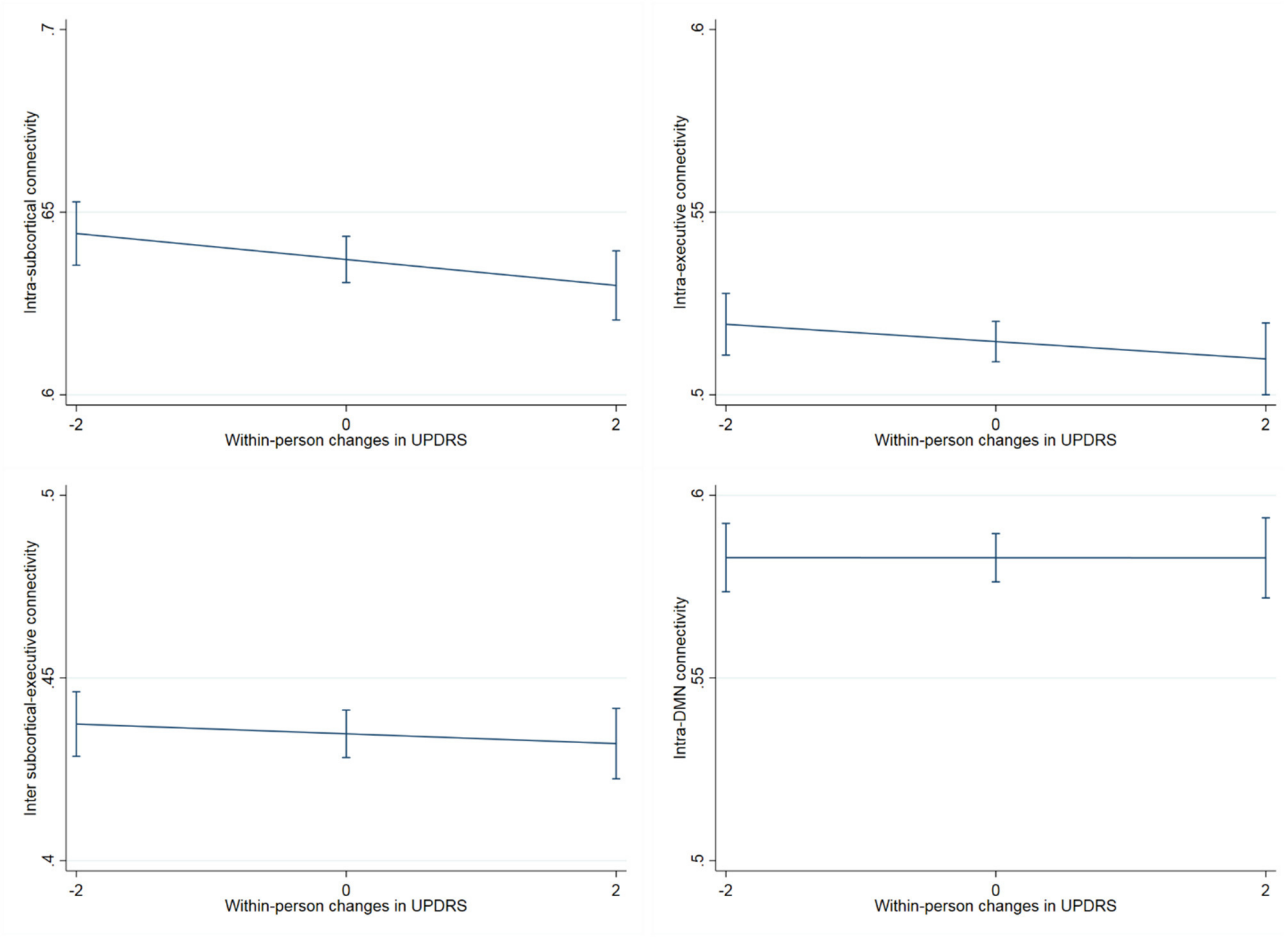
Association between within-person changes in UPDRS scores and functional network connectivity. The figure shows within-person increases in UPDRS scores significantly associated with reduced synchrony of intra-subcortical network connectivity over time (*b* = −0.0036, *p* = 0.029), this effect additionally held when subcortical network gray matter volume was adjusted for (*b* = −0.0035, *p* = 0.035). On the other hand, the relationship between UPDRS and intra-frontoparietal executive network connectivity and/or inter subcortical- frontoparietal network connectivity did not show significant within-person effects. The presumably unrelated DMN network, was also not significantly associated with UPDRS scores over time.

Regarding neural connectivity, we demonstrated that within-person increases in UPDRS scores were significantly associated with reduced within-person synchrony of intra-subcortical network connectivity over time (UPDRS within-person *b* = −0.0036, *p* = 0.029; UPDRS between-person *b* = −0.0003, *p* = 0.83) (Figure 5), this effect additionally held when subcortical network gray matter volume was adjusted for (UPDRS within-person *b* = −0.0035, *p* = 0.035). Models examining the relationship between UPDRS and intra-frontoparietal executive network connectivity were not statistically significant (UPDRS within-person *b* = −0.002, *p* = 0.21; UPDRS between-person *b* = −0.0003, *p* = 0.83). To further demonstrate specificity of the subcortical effect, we examined a presumably unrelated network measure, DMN connectivity, which also was not significantly associated with UPDRS scores over time (UPDRS within-person *b* = −0.0001, *p* = 0.970; UPDRS between-person *b* = −0.0019, *p* = 0.220) (Table 3). We additionally adjusted for WM integrity using FA of the corpus callosum and the primary effects remained.

**Table 3:** Within person and Between person UPDRS scores and fMRI network connectivity correlations in a cohort of functionally intact community-dwelling older adults.

	**Sample size** **(number of observations)**	**Within person**		**Between person**	
		**Coefficient (95% CI)**	***p*-Value**	**Coefficient (95% CI)**	***p*-Value**
Intra subcortical network connectivity	330 (546)	0.0036 (−0.0068, −0.0004)	0.029	0.0003 (−0.0032, 0.0026)	0.828
Intra fronto-parietal executive network connectivity	330 (546)	−0.002 (−0.005, 0.0011)	0.211	0.0003 (−0.0022, 0.0028)	0.826
DMN connectivity	330 (546)	−0.0001 (−0.0035, 0.0034)	0.970	−0.0019 (−0.0049, 0.0011)	0.220

*Within person and between person coefficients [and 95% Confidence Interval (CI)] are shown. Covariates in all models included baseline age, sex, and education, and mixed effects models allowed for subject specific intercepts and slopes. Within-person increases in UPDRS scores were significantly associated with reduced within-person synchrony of intra-subcortical network connectivity over time. DMN stands for Default Mode Network*.

## Discussion

The aim of this study was to characterize the longitudinal nature of Parkinsonian signs, including their cognitive and neural (via functional connectivity) correlates, among functionally normal older adults. The data we collected represents the longest and largest cohort of functionally intact participants with subclinical UPDRS measurements. Our data demonstrates that UPDRS scores increase in functionally intact aging adults, particularly in those >60 years old, and even minimal motor symptom increases appear to meaningfully track with reductions in processing speed and intra-subcortical network synchrony over time.

Our finding that relative changes within an individual relate to both, a pattern of slowed processing speed, as well as changes in a subcortical network that includes the basal ganglia, underscores the potential importance of these motor signs (Shulman et al., 2010), which can be detectable among “subclinical” older adults. On the other hand, we also examined relationships between UPDRS scores and executive functions and mood. In this case, UPDRS showed an overall relationship (between person effect), but not a within-person effect; these data may suggest that overall severity of Parkinsonian signs relate to overall executive functioning and mood *traits*, but are less sensitive to within-person changes at least in this subclinical range.

Regarding neural correlates, we showed that within-person increases in UPDRS scores among functionally intact elders, are significantly associated with reduced synchrony of intra-subcortical network connectivity over time. Between-person effects shows the overall influence (average) association between the UPDRS and fMRI outcomes, while the within-person effect shows the effect relationship between the changes in the UPDRS from visit to visit and fMRI outcomes. It is possible that there is also a between person effect that is smaller in size and is overshadowed by the within-person effect in the models. The fact that only the latter is significant explains why we did not see a cross-sectional effect. Perhaps, if we follow these population for a longer period of time, we would also be able to see a relatively smaller between-person effect. Given this is a “subclinical” cohort, it may not be surprising that overall UPDRS scores were low with less variance compared to a clinical cohort and therefore overall UPDRS scores did not relate as strongly to our outcomes (i.e., restricted range).

Although the neural correlates of Parkinsonism are well-characterized in PD, little is known about the neural underpinnings of Parkinsonism in healthy older adults. In PD, task-free fMRI studies have generally found reduced connectivity globally, within the striatum, or between the striatum and other areas, including the globus pallidus, thalamus, midbrain, upper pons, cerebellum, and other regions (Hacker et al., 2012; Wu et al., 2012; Rolinski et al., 2015). Furthermore, these studies in PD have typically found that connectivity between the striatum correlates with the UPDRS (Tuovinen et al., 2018), although one study by Szewczyk-Krolikowski did not replicate this finding (Szewczyk-Krolikowski et al., 2014). More recently, Wang et al. (2018) utilized the UPDRS score to quantify motor signs in PD, and they also found that functional connectivity was positively correlated with Parkinsonism using this measure. All of these studies were performed on PD cohorts, however, our study adds to these literature by finding a correlation between UPDRS sub-clinical scores and reduced within-person synchrony of intra-subcortical network connectivity over time, in a non-PD cohort.

We also observed associations between UPDRS scores and depressive symptoms. Although it is understood that normal aging can be associated with developing depression, the biochemical or morphological basis for this is not known. Since we found an overall effect of UPDRS on mood, it is possible that the motor signs operate in conjunction with mood, exacerbating or causing mood disturbances or that vascular changes typical of aging affect both the motor and mood centers of the brain. Another parsimonious explanation might be that both changes are related to reduced neurotransmitter function secondary to aging; both serotonin and norepinephrine are intimately involved in mood. Furthermore, they serve in several brain regions involved in movement and motor activity. Additionally, they are known to decline with aging, as well as in AD and PD patients, interestingly, brainstem norepinephrine neurons have been said to degenerate prior to the cholinergic neurons in AD and prior to SN dopamine neurons in PD (Zarow et al., 2003). Future work is needed in order to address these questions of causes and mechanisms.

Parkinsonism and gait decline are common among older adults and are a risk factor for adverse outcomes (Yuan et al., 2015). Such age-related gait decline has been widely studied and repeatedly shown to increase the risk for morbidity, hospitalization, and mortality (Newman et al., 2006; Verghese et al., 2006; Studenski et al., 2011). It is also well-established that performances on motor tasks correlates with declining cognition in the healthy population (Atkinson et al., 2010; Demnitz et al., 2016), and specifically, age-related declines in gait may be a risk for future cognitive decline and dementia in older adults (Yuan et al., 2015; Jor'dan et al., 2017). Interestingly, Mielke et al. (2013) found a slow gait was associated with decline in executive functioning. Our study, to our knowledge, is the first to leverage an easy and replicable measuring tool, the UPDRS, in order to show this association between Parkinsonism motor symptoms and cognitive performance in a functionally intact population. It is also the first one to study these changes not cross-sectionally, but longitudinally. We would highly encourage other studies performing research on the healthy population to consider collecting UPDRS scores and replicating these and other associations with the hopes of truly understanding their significance.

Several candidate patho-physiological mechanisms may explain the association between aging and Parkinsonian changes: First, Parkinsonian signs can be a consequence of early protein deposition, either alpha-synuclein or amyloid. Second, vascular events, such as cerebral infarction or small vascular diseases (leukoaraiosis, amyloid angiopathy, and microbleeds). Third, they could be the result of calcification in the striatum. Fourth, iron deposition, which although is usually assumed to be “benign,” recently Salami et al. proposed that striatal iron content was negatively associated with the number of connections and the spontaneous coherence within the caudate and putamen resting state networks; suggesting that iron can modulate the degree of connections between the striatum and cerebral cortex -importantly, these associations were primarily driven by the older group. Finally, in line with our results, they found a positive correlation between coherence in the putamen and motor performance, suggesting that the decreased spontaneous activity secondary to iron deposition has behavioral consequences (Salami et al., 2018).

Another potential contributor to Parkinsonism in healthy older adults is a decline in dopamine transmission level that occurs in the aging brain (Kaasinen and Rinne, 2002). Autopsy and molecular imaging studies have identified that the causes of age-associated decrease in dopamine transmission are multi-factorial (Kaasinen and Rinne, 2002). Older adults have a lower absolute level of dopamine (Garnett et al., 1983), dopamine receptors (Suhara et al., 1991; Kaasinen et al., 2000; Inoue et al., 2001), and dopamine transporters when compared to young adults (Volkow et al., 1994; van Dyck et al., 1995; Rinne et al., 1998). Due to this decrease in dopamine transmission, the aging brain could be considered to be on the preclinical continuum of Parkinson's disease. Dopamine also plays a role in cognitive functions, such as working memory (Arnsten and Li, 2005). Several studies have associated the level of dopamine receptor or transporter density and executive function and working memory in older adults (Backman et al., 2000; Mozley et al., 2001). For this study, we did not have direct measures of dopaminergic function available, but this may be an important direction for future research.

These data, however, are not without limitations. First, given that the longitudinal data collection started in 2008, we used the original UPDRS instead of the newly validated MDS-UPDRS. Second, UPDRS is performed by physicians and can be a somewhat subjective measure, with some items like speech, facial expression, posture, body bradykinesia, action tremor, and rigidity having a relatively poor inter-rater reliability (Martinez-Martin et al., 1994; Richards et al., 1994). Third, though we were interested in which specific UPDRS items were driving this correlation, given that our observed UPDRS scores were relatively low overall in this functionally intact cohort, we did not find any signs (tremor or slowness related) to be more prominent than others (i.e., overall low variability). Fourth, it is possible that there are non-linear effects, but because the average number of visits was only two, we were limited in our ability to test non-linear terms. Fifth, UPDRS scores can become elevated due to other causes that are not related to the central nervous system, such as arthritis; these data were not routinely collected and therefore were not accounted for in our analyzes. Nonetheless, we found an association between observed motor signs and other indicators of central nervous system functioning (fMRI and cognition), suggesting that these signs are likely not wholly accounted for by peripheral disease. Sixth, our population is a fairly homogeneous sample of highly educated, white participants who are not representative of all older adults, so it is not clear how much these results can be generalized to other populations. Lastly, some of our study participants use medications that could have confounded our results, including psychoactive medication, such as benzodiazepines or betablockers; both diminish tremor and could have lowered participants' UPDRS scores.

In conclusion, our findings that subclinical motor changes are associated with declines in cognitive, mood, and neural function, extends the typical aging literature by indicating that motor signs may function along a continuum. Similar to how mild cognitive impairment (MCI) represents the stage of cognitive decline between normal aging and dementia, mild Parkinsonian signs could potentially represent the mild end of a disease spectrum that spans from normal aging to a Parkinsonian disease. Importantly, just as MCI does not indicate that an individual will get dementia, these Parkinsonian signs do not necessarily imply that a person will develop PD. Other potential etiologies include vascular changes or protein depositions that occur as part of the aging process. Even in our cohort of community-dwelling adults, these motor signs tracked closely with markers of brain and cognitive health. Thus, given that the UPDRS is an inexpensive test that can be easily incorporated in the routine examination of elderly persons, this motor rating scale may potentially not only be useful in patients with Parkinsonian disorders, but for screening of the elderly population in general to be used along with measures of cognition to identify heightened risk for future neurodegeneration among initially healthy older women and men.

## Data Availability Statement

The raw data supporting the conclusions of this article will be made available by the authors, without undue reservation.

## Ethics Statement

The University of California San Francisco Institutional Review Board (IRB) approved this study and informed written consent was obtained from all participants.

## Disclosure

CS is now a full-time employee at the Alzheimer's Association. BM receives grants in support of the Memory and Aging Center from the NIH/NIA, the Quest Diagnostics Dementia Pathway Collaboration, Cornell University and The Bluefield Project to Cure Frontotemporal Dementia. BM serves as Medical Director for the John Douglas French Foundation; Scientific Director for the Tau Consortium; Director/Medical Advisory Board of the Larry L. Hillblom Foundation; and Past President of the International Society of Frontotemporal Dementia (ISFTD). JK is on a Biogen advisory board.

## Author Contributions

JZ and KC conceived the presented idea. KC verified the analytical methods and statistics. fMRI methods and analysis were performed by AS and JB. JZ wrote the manuscript. All authors discussed the results and contributed to the final manuscript.

## Conflict of Interest

The authors declare that the research was conducted in the absence of any commercial or financial relationships that could be construed as a potential conflict of interest.
