# Supplementary material for: Mild Motor Signs Matter in Typical Brain Aging: The Value of the UPDRS Score Within a Functionally Intact Cohort of Older Adults
Source: Front Aging Neurosci. 2021 Feb 11;13:594637. doi: 10.3389/fnagi.2021.594637 (PMC7904682; doi:10.3389/fnagi.2021.594637)
Supplement: Supplementary file 1 [file Presentation_4.pdf]

Supplementary material: Text S1

Volumetric Image Processing

Before processing, all T1-weighted images were visually inspected for quality. Images with excessive motion or image artifact were excluded. Magnetic field bias was corrected using the N3 algorithm.(Sled et al., 1998) Tissue segmentation was performed using the unified segmentation procedure in SPM12.(Ashburner and Friston, 2005) Each participant's T1-weighted image was warped to create a study-specific template using Diffeomorphic Anatomical Registration using Exponentiated Lie algebra (DARTEL);(Ashburner, 2007) subsequently, the images were normalized and modulated in the study-specific template space using non-linear and rigid-body registration. Images were smoothed using an 8mm Gaussian kernel with 8-mm full width half maximum. For registration with a brain parcellation atlas, linear and non-linear transformations between DARTEL's space and International Consortium of Brain Mapping (ICBM) space were applied.(Mazziotta et al., 1995) Quantification of volumes in specific brain regions at each time point was accomplished by transforming a standard parcellation atlas into ICBM space and summing all modulated gray matter within each parcellated region.(Desikan et al., 2006) Total Intracranial Volume (TIV) was estimated for each subject in MNI space.(Malone et al., 2015) Gray matter volume (GMV) and TIV are reported in cm^3.

Supplementary material: Text S2

fMRI Preprocessing

For each fMRI scan, the first five volumes were discarded. SPM12 (http://www.fil.ion.ucl.ac.uk/spm/software/spm12/) and FSL (http://fsl.fmrib.ox.ac.uk/fsl) software was used for subsequent fMRI preprocessing. The remaining 235 volumes were slice-time corrected, realigned to the mean functional image and assessed for rotational and translational head motion. Volumes were next co-registered to the MP-RAGE image, then normalized to the standard MNI-152 healthy adult brain template using SPM segment, producing MNI-registered volumes with 2 mm^3 isotropic resolution. These volumes were spatially smoothed with a 6-mm radius Gaussian kernel and temporally bandpass filtered in the 0.008-0.15Hz frequency range using fslmaths. Nuisance parameters in the preprocessed data were estimated for the CSF using a mask in the central portion of the lateral ventricles and for the white matter using a mask of the highest probability cortical white matter as labeled in the FSL tissue prior mask. Additional nuisance parameters included the 3 translational and 3 rotational motion parameters, the temporal derivatives of the previous 8 terms (WM/CSF/6 motion), and the squares of the previous 16 terms.(Satterthwaite et al., 2013) Subjects were included only if they met all of the following criteria: no inter-frame head translations greater than 3 mm, no inter-frame head rotations greater than 3 degrees, and less than 24 motion spikes (defined as inter-frame head displacements > 1 mm), 10% of the total number of frames.

Those excluded nodes that were part of the DMN networks were: left frontal medial cortex (47), right frontal pole (48), left inferior (95) and middle (81) temporal gyri, posterior divisions.

Excluded nodes from the frontoparietal network included posterior, temporal gyrus (99, 101), the right frontal pole (46), and right inferior temporal gyrus (100, 102). Numbers in parentheses correspond to the nodes in the Brainnetome atlas. No subcortical nodes were excluded.

References:

Ashburner, J., 2007. A fast diffeomorphic image registration algorithm. NeuroImage 38(1), 95-113.

Ashburner, J., Friston, K.J., 2005. Unified segmentation. NeuroImage 26(3), 839-851.

Desikan, R.S., Segonne, F., Fischl, B., Quinn, B.T., Dickerson, B.C., Blacker, D., Buckner, R.L., Dale, A.M., Maguire, R.P., Hyman, B.T., Albert, M.S., Killiany, R.J., 2006. An automated labeling system for subdividing the human cerebral cortex on MRI scans into gyral based regions of interest. NeuroImage 31(3), 968-980.

Malone, I.B., Leung, K.K., Clegg, S., Barnes, J., Whitwell, J.L., Ashburner, J., Fox, N.C., Ridgway, G.R., 2015. Accurate automatic estimation of total intracranial volume: a nuisance variable with less nuisance. NeuroImage 104, 366-372.

Mazziotta, J.C., Toga, A.W., Evans, A., Fox, P., Lancaster, J., 1995. A probabilistic atlas of the human brain: theory and rationale for its development. The International Consortium for Brain Mapping (ICBM). NeuroImage 2(2), 89-101.

Satterthwaite, T.D., Elliott, M.A., Gerraty, R.T., Ruparel, K., Loughead, J., Calkins, M.E., Eickhoff, S.B., Hakonarson, H., Gur, R.C., Gur, R.E., Wolf, D.H., 2013. An improved framework for confound regression and filtering for control of motion artifact in the preprocessing of resting-state functional connectivity data. NeuroImage 64, 240-256.

Sled, J.G., Zijdenbos, A.P., Evans, A.C., 1998. A nonparametric method for automatic correction of intensity nonuniformity in MRI data. IEEE transactions on medical imaging 17(1), 87-97.
